# An immortalized porcine macrophage cell line competent for the isolation of African swine fever virus

**DOI:** 10.1038/s41598-021-84237-2

**Published:** 2021-02-26

**Authors:** Kentaro Masujin, Tomoya Kitamura, Ken -ichiro Kameyama, Kota Okadera, Tatsuya Nishi, Takato Takenouchi, Hiroshi Kitani, Takehiro Kokuho

**Affiliations:** 1 African Swine Fever Unit, National Institute of Animal Health, National Agriculture and Food Research Organization (NARO), 6-20-1 Josuihoncho, Kodaira, Tokyo Japan; 2 Foot and Mouth Disease Unit, National Institute of Animal Health, National Agriculture and Food Research Organization (NARO), 6-20-1 Josuihoncho, Kodaira, Tokyo Japan; 3 Division of Animal Sciences, Institute of Agrobiological Sciences, NARO, 1-2 Ohwashi, Tsukuba, Ibaraki Japan

**Keywords:** Infectious-disease diagnostics, Virology

## Abstract

African swine fever virus (ASFV) is the etiological agent of African swine fever (ASF), a fatal hemorrhagic disease of domestic pigs and wild boar. The virus primarily infects macrophage and monocyte host cells, these do not grow in vitro. Many attempts have been made to establish sustainable ASFV-sensitive cell lines, but which supported only low viral replication levels of limited, mostly artificially attenuated strains of ASFV. Here, we examined the competence of a novel cell line of immortalized porcine kidney macrophages (IPKM) for ASFV infection. We demonstrated that IPKM cells can facilitate high levels (> 10^7.0^ TCID_50_/mL) of viral replication of ASFV, and hemadsorption reactions and cytopathic effects were observed as with porcine alveolar macrophages when inoculated with virulent field isolates: Armenia07, Kenya05/Tk-1, and Espana75. These results suggested that IPKM may be a valuable tool for the isolation, replication, and genetic manipulation of ASFV in both basic and applied ASF research.

## Introduction

African swine fever (ASF) is a highly lethal hemorrhagic disease of domestic pigs and wild boar (*Sus scrofa*). ASF, which is endemic to sub-Saharan Africa and Sardinia^1^, was recently and accidently introduced into Georgia in 2007, and then spread into Russia and Central Europe^2–4^. In 2018, ASF reached China^5^, the largest pork producer in the world, and is currently circulating in Asia and the Pacific region^6^. The etiological agent, African swine fever virus (ASFV), is maintained among natural reservoirs of African wild suid species, such as common warthogs (*Phacochoerus africanus*), bush pigs (*Potamochoerus larvatus*) and *Ornithodoros* ticks^7^. While these wild mammalian species do not show any clinical signs after ASFV infection, infections of domestic pigs and wild boar are usually accompanied by peracute to chronic symptoms after onset, with high case fatality rate^8^. The symptoms of ASFV, currently circulating in Europe and Asia, are mostly peracute to acute types. Subacute disease progression is only sometimes described (e.g. from Trans Caucasus), while chronic disease is mostly described historically from the Iberian Peninsula^9,10^. ASFV is the only member of the family *Asfarviridae*, genus *Asfivirus*, and is morphologically and biologically distinct from other mammalian viruses^11^. The virion has a five-layered structure, is 260–300 nm in size, and harbors 170–190 kbp long double-stranded genomic DNA in the nucleoid core. This large genome contains more than 170 open reading frames (ORFs) that encode genes that facilitate the efficient replication of the virus in host cells^12^.

Since ASFV is presently known to primarily infect macrophages and monocytes^8,13^ numerous efforts have been made to establish immortalized cell lines of these lineages to aid investigations of the biological properties of the virus (i.e., replication cycle, host immune modulation, and pathogenesis) and thus develop specific diagnostic systems, antiviral drugs, and efficacious vaccine candidates. Recently, the authors of the present study established a novel immortalized cell line from primary porcine kidney macrophages by introducing SV40 large T antigen (SV40LT) and porcine telomerase reverse transcriptase (pTERT) genes with modified lentiviral vectors^14^. This established cell line, IPKM (immortalized porcine kidney macrophages), expresses a set of macrophage-specific differentiation markers, such as Iba1, KT022, and CD172a, endocytoses exogenous microparticles, and produces TNFα and IL-1β at levels comparable to those of primary porcine kidney macrophages after lipopolysaccharide stimulation, indicating an intermediate- to late-macrophage-like phenotype that is expected to be susceptible to ASFV infection^14^. In the present study, we examined the competency of the IPKM cell line for ASFV infection and evaluated its suitability for a wide range of in vitro ASFV studies.

## Results

### Susceptibility of IPKM to ASFV isolates

**Figure 1:**
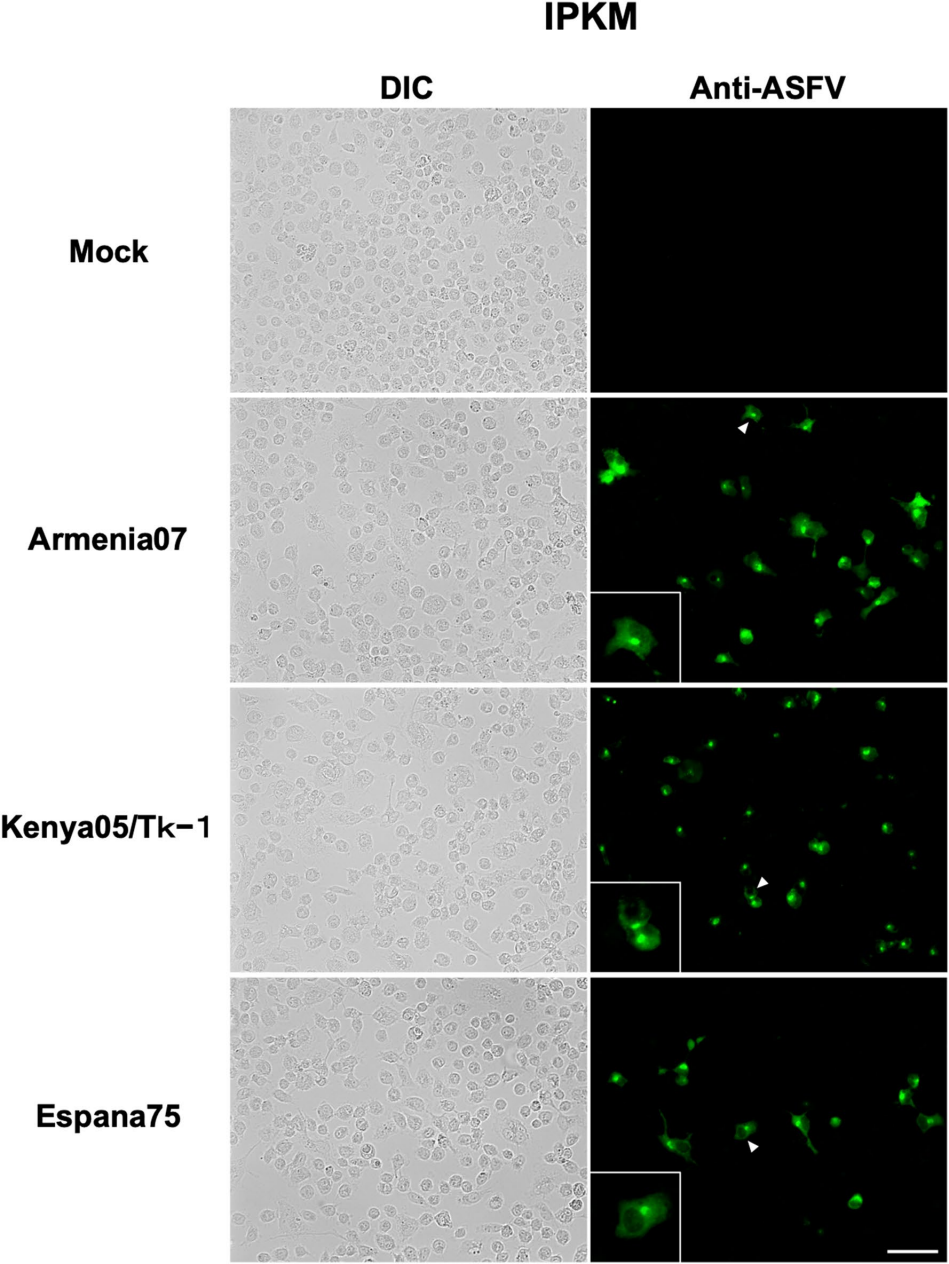
Immunofluorescence assay in immortalized porcine kidney macrophages (IPKM). IPKM cell cultures were mock-inoculated or inoculated with Armina07, Kenya05/Tk-1, and Espana75 isolates (MOI = 0.1), fixed at 16 h post inoculation, and incubated with FITC-conjugated anti-ASFV antibodies to detect the viral particles. The left and right columns show differential interference contrast (DIC) and fluorescence images, respectively. The images are representatives from five independent experiments. The inserts in the lower left corners of the fluorescence images are magnified images of the cells, indicated by arrowheads in their corresponding panels. Bar = 50 µm.

To examine the susceptibility of the IPKM cell line to ASFV infection, IPKM cells were inoculated with virulent ASFV field isolates, Armenia07 (genotype II), Kenya05/Tk-1 (genotype X), and Espana75 (genotype I). Although cytopathic effects (CPEs) were not clearly observed at the time of fixation (16 h post-inoculation; hpi), fluorescent signals were detected at the perinuclear areas of the IPKM cells inoculated with all of the isolates listed above via immunofluorescent staining with a monoclonal antibody (mAb) against ASFV. Fluorescent signals were not detected from mock-infected control cells (Fig. 1). These results indicated that IPKM is susceptible to infection by virulent field isolates of ASFV.

### Detection of ASFV in IPKM cell cultures

**Figure 2:**
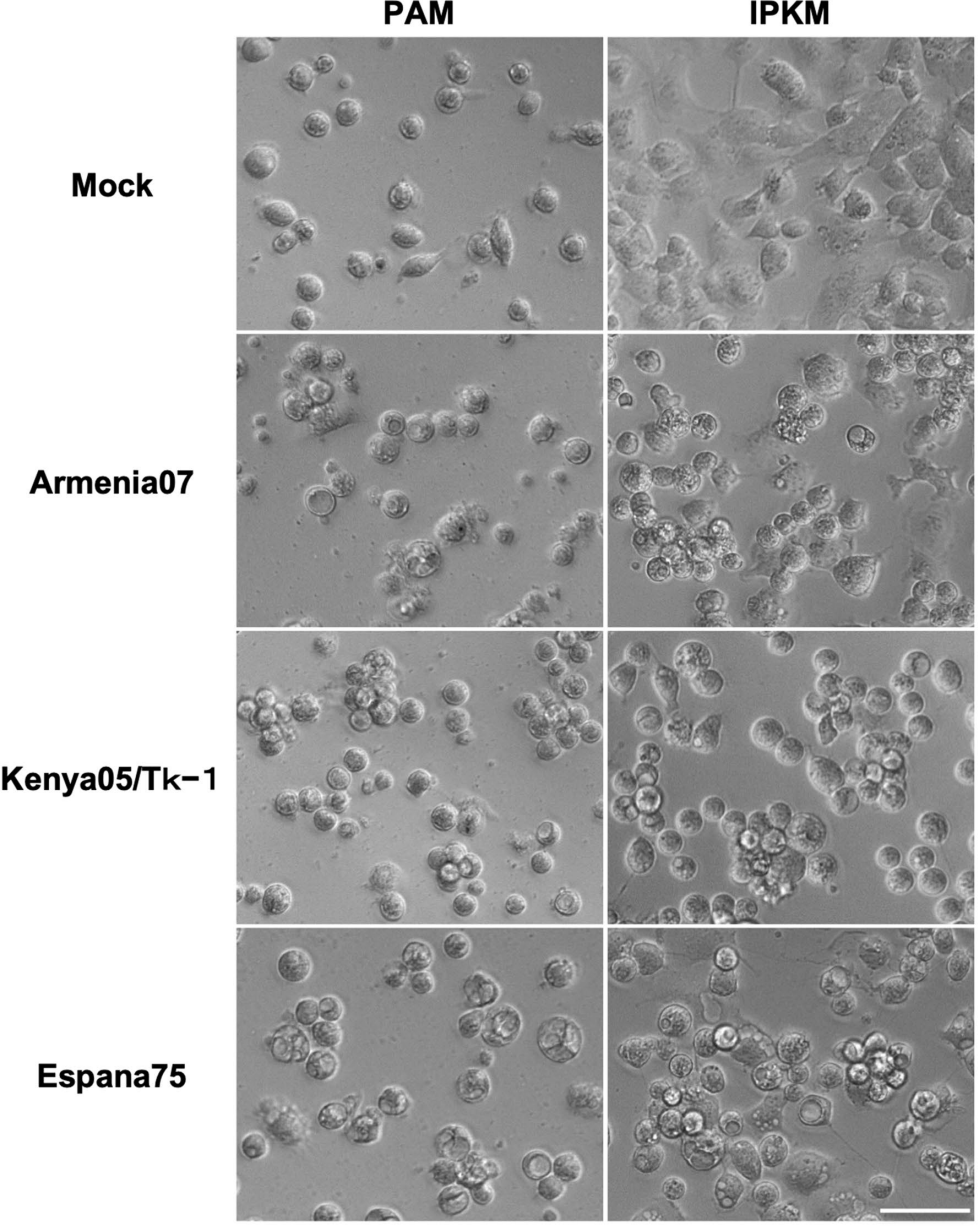
Cytopathic effects in primary porcine alveolar macrophages (PAM) and immortalized porcine kidney macrophages (IPKM). Cell cultures were mock-inoculated or inoculated with Armenia07, Kenya05/Tk-1 and Espana75 isolates (MOI = 0.1). The left and right columns show the PAM and IPKM cell lines at 3 days after inoculation, respectively. Images are representatives from three experiments that demonstrated similar trends. Bar = 50 µm.

**Figure 3:**
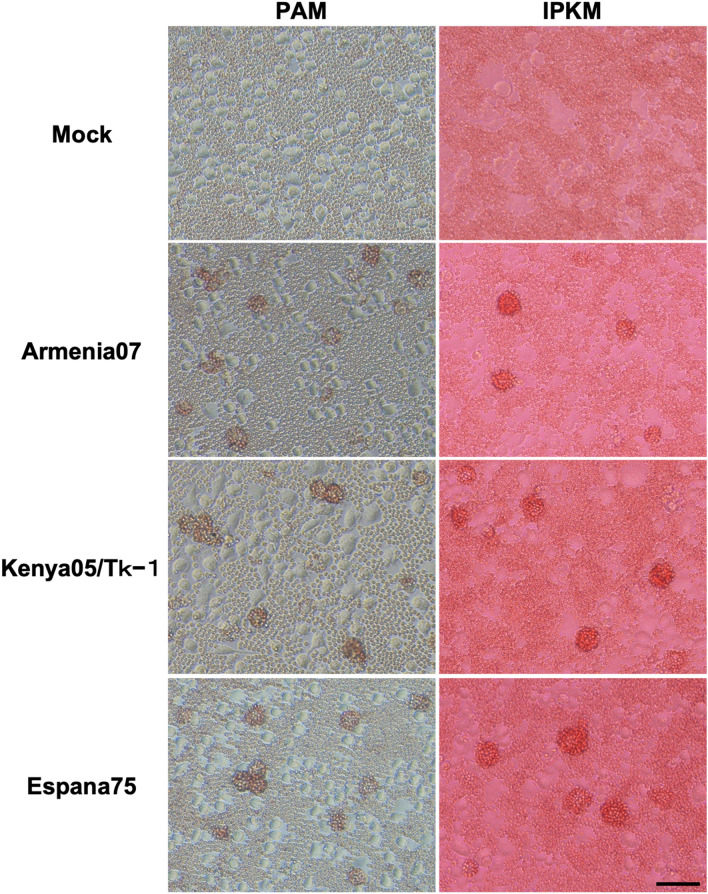
Hemadsorption assay in primary porcine alveolar macrophages (PAM) and immortalized porcine kidney macrophages (IPKM). Cell cultures were mock-inoculated or inoculated with Armina07, Kenya05/Tk-1 and Espana75 isolates (MOI = 0.1) in the presence of porcine red blood cells. The left and right columns show the PAM and IPKM cell cultures at 1 day after inoculation, respectively. The images are representatives from five independent experiments. Bar = 50 µm.

We performed virus propagation assays with primary porcine alveolar macrophages (PAM) and IPKM cell cultures to examine if IPKM cells can support the replication of infectious ASFVs. With viral inoculations at a multiplicity of infection (MOI) of 0.1, the Armenia07 and Kenya05/Tk-1 isolates induced CPEs in the IPKM cell cultures at 2 days post-inoculation (dpi), while the Espana75 isolate induced CPEs at 3 dpi. ASF-infected IPKM cells became round in shape, formed grape-like clusters of 3–20 cells, then, detached from the bottom of a cell culture plate as CPEs developed (Fig. 2). All three isolates showed CPEs at 3 dpi in the PAM cultures (Fig. 2). The appearance of ASFV-infected PAM cells was similar to that of ASFV-infected IPKM cells, however, ASFV-infected cells developed less clear CPEs than ASFV-infected IPKM cells (Fig. 2). In the hemadsorption (HAD) assays, a rosette formation was confirmed in both IPKM and PAM cell cultures inoculated with the tested ASFV isolates but not in mock-infected controls at 1 dpi in the presence of porcine red blood cells (Fig. 3).

### Viral growth in IPKM cell cultures

**Figure 4:**
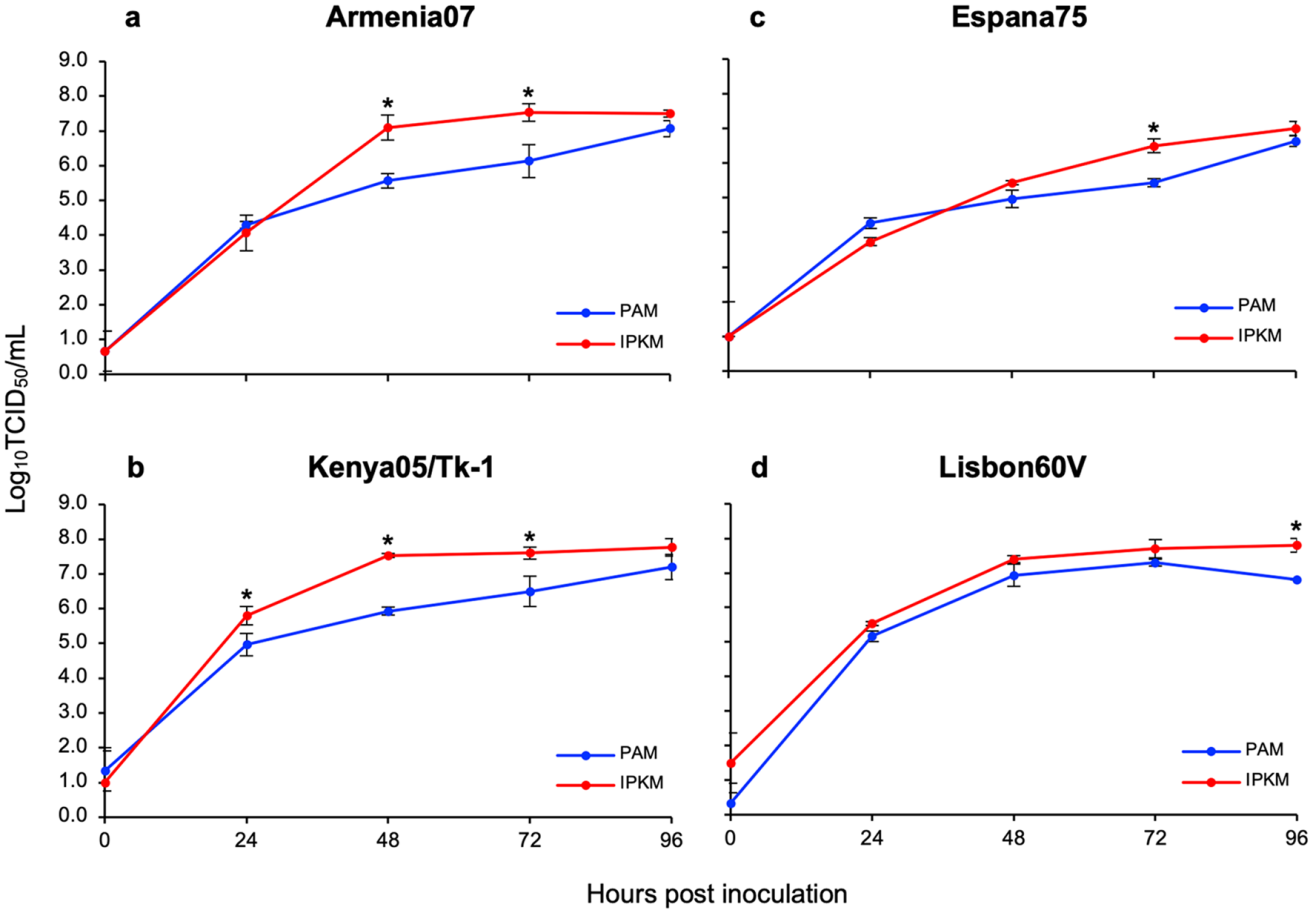
Comparison of the African swine fever virus (ASFV) production in primary porcine alveolar macrophages (PAM) and immortalized porcine kidney macrophages (IPKM). Cell cultures were infected with Armina07 (**a**), Kenya05/Tk-1 (**b**), Espana75 (**c**) and Lisobon60V isolates (**d**) (MOI = 0.001). The culture supernatant was recovered at the indicated time-point infection. Viral production in the PAM and IPKM cell cultures were estimated by titration. Data represent the means and standard deviations of three experiments. Asterisks indicate statistically significant differences in viral production in the PAM and IPKM cell cultures (*p* < 0.05, Student’s *t*-test).

To further evaluate the potential of the IPKM cell culture system in the virus titration assays, we compared the viral titers produced in the IPKM and PAM cell cultures over time (0–96 hpi) (Fig. 4). The Armenia07 and Kenya05/Tk-1 isolates were propagated in IPKM cell cultures and reached maximal titers of 10^7.1^ and 10^7.5^ TCID_50_/mL at 48 hpi, respectively. The viral titers remained stable thereafter, at least up until 96 hpi. In contrast, both isolates propagated slowly in PAM cells and at 48 and 72 hpi they exhibited 30–40 times lower viral titers compared to those of IPKM cells. These differences became negligible at the end of culture period (96 hpi) (Fig. 4a,b). The Espana75 isolate also replicated similarly, but the difference in viral titers between PAM and IPKM cells at 48 hpi was not obvious. The maximal titer (10^7.0^ TCID_50_/mL) of the Espana75 isolate at 96 hpi was slightly lower than that of the other two isolates (Fig. 4c).

Next, we attempted to assess and compare the propagation of a cell-adapted isolate of ASFV in the IPKM and PAM cell cultures. The Vero cell-adapted isolate of Lisbon60 strain (genotype I), Lisbon60V, which was established by 33 repetitive passages in Vero cell cultures, was inoculated into the IPKM and PAM cell cultures at a MOI of 0.001. The Lisbon60V isolate replicated in a similar manner in the IPKM and PAM cell cultures. The Lisbon60V isolate reached peak levels at 72 hpi, but a slight reduction in the viral titer was observed in the PAM cell culture at 96 hpi (Fig. 4d).

### Virus purification in IPKM cell cultures

**Figure 5:**
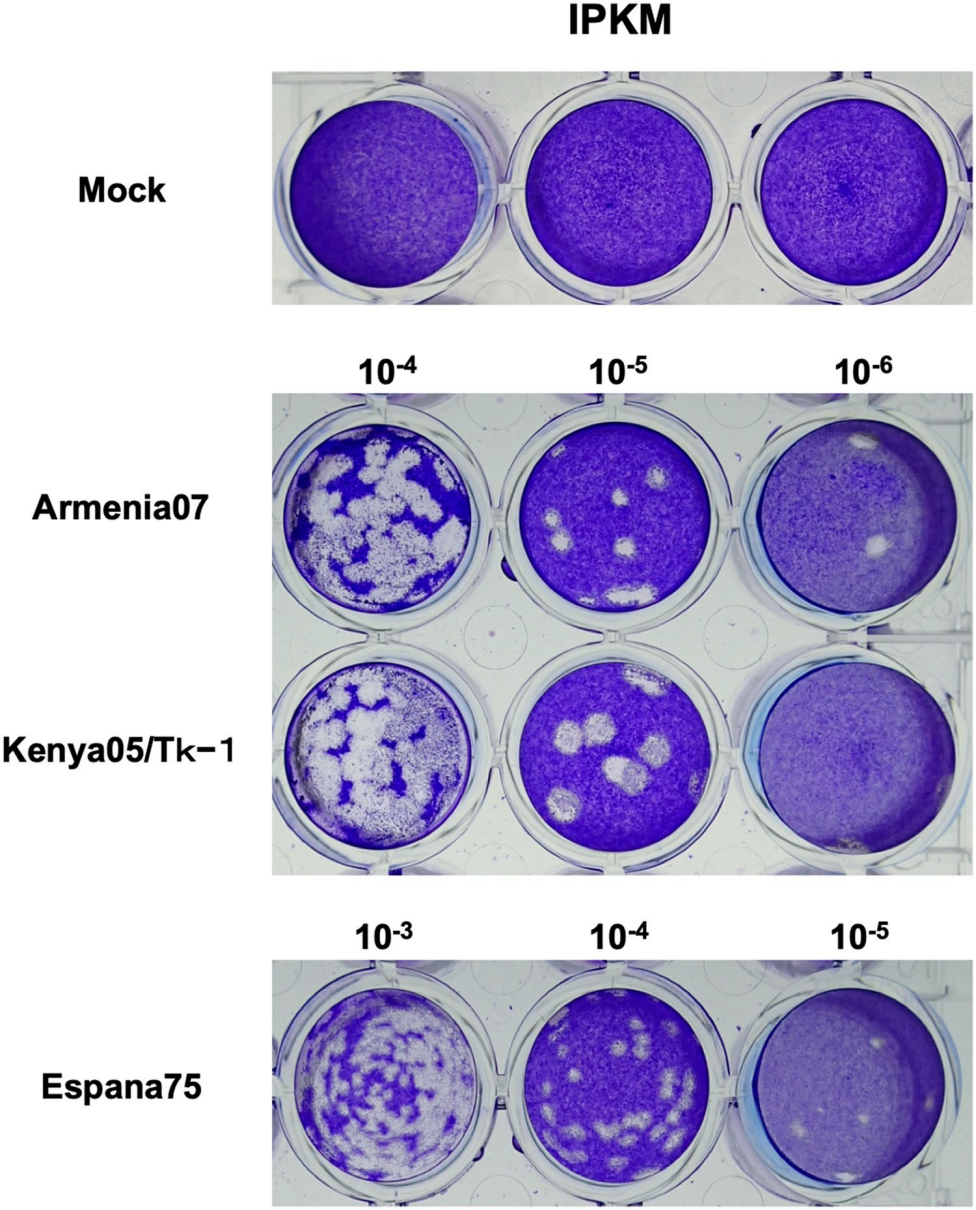
Plaque assay of African swine fever virus (ASFV) in immortalized porcine kidney macrophages (IPKM). IPKM cells cultured in 24-well cell culture plates were inoculated with tenfold dilutions of ASFV isolates (Armenia07, Kenya05/Tk-1 and Espana75), overlaid with agar medium and incubated, before staining with crystal violet.

We examined the suitability of IPKM cell cultures for in vitro virus isolation and purification. As shown in Figs. 2 and 5, ASFV-infected IPKM cells developed clear CPEs in liquid cultures and formed sharply defined plaques in agarose layered, crystal violet stained cultures in a dose-dependent manner. PAM cells also exhibited CPEs, but the shapes of the plaques made it too difficult to count the number of plaques formed per well and isolate a plaque of interest from neighboring ones (data not shown). Interestingly, the size of the plaques was isolate-dependent. As shown in Fig. 5, the Kenya05/Tk-1 isolate and the Espana75 isolate had the largest and the smallest plaque sizes, respectively, in the IPKM cell cultures.

### Frequency of genetic changes in the ASFV genome by repeated passages in IPKM

Genetic alterations may occur during the adaptation process of ASFV^15–18^. Thus, we attempted to evaluate the effect of passage in the IPKM cell cultures on the occurrence of spontaneous mutations in the genome. We conducted genome-wide comparative analysis of the next-generation sequencing data obtained from Armenia07 isolates at passage levels of 0, 5, 10, and 15 in IPKM cell cultures, and found that only one non-synonymous nucleotide replacement (C to T) occurred at the position of 125,911 in the CP530R region of the viral genome at passage levels 10 and 15 (Table 1).

**Table 1:** A mutation accumulated in the genome DNA of ASFV Armenia07 isolate by serial passages in IPKM cells.

Position^a^	Gene	Type of modification	Number of passages (P) and mutation			
			P0	P5	P10	P15
125,911	CP530R^b^	C-to-T, Ser169Lue	C	C	T	T

^a^Nucleotide position number is based on the sequence of the ASFV Georgia 2007/1 isolate [GenBank accession no. FR682468].

^b^The CP530R encodes polyprotein, pp60, which is cleaved to p35 and p15. These proteins form the major components of the core shell of the virion.

## Discussion

Primary cultures of porcine macrophages and monocytes derived from peripheral blood or various tissues, including alveolar macrophages, are the only available in vitro systems suitable for the detection, isolation, and genetic manipulation of field isolates of virulent ASFV. These systems are useful for a wide range of ASF studies in combination with various techniques, such as HAD assays and biological staining. However, the quality of primary cell preparations may vary among batches, for example, as a result of differences in the health status of donor animals and preparation skills. Such variabilities in culture cell conditions may occasionally impede research progress in this field; hence, sustainable cell lines that are susceptible to the infection of ASFV are in great demand. In this study, we demonstrated the susceptibility of a recently established porcine kidney macrophage cell line, IPKM, to the infection of ASFV field isolates and Vero cell-adapted viruses of different genotypes. The present data strongly suggest that IPKM cell culture systems are highly suitable for efficient isolation, propagation, and genetic modification of both virulent and cell-adapted isolates of ASFV.

IPKM cells developed clear CPEs when infected with ASFV. Based on this characteristic property, we established an assay suitable for virus titration and confirmed that the detection limit of the assay was almost identical to that of the PAM-based classical HAD assay (data not shown). IPKM is an immortalized cell line, which can be routinely maintained at a laboratory at low cost; therefore, this cell line could be an ideal tool for diagnosis of ASF in the case of an outbreak. We also demonstrated that IPKM cells were useful for plaque forming assays of ASFV, which enabled the rapid isolation and purification of both wild-type and genetically modified viruses. Interestingly, in plaque forming assay using IPKM cell cultures, the sizes of plaque produced by ASFV varied isolate-dependently. In general, the difference in plaque size reflects viral fitness, such as replication rate, transmission efficiency and capacity of host immune evasion in a given culture system^19–21^. In the present data, the Espana75 isolate, which replicated at a slower rate with a lower titer compared to Armenia07 and Kenya05/Tk-1 strains, demonstrated small plaque phenotype in the IPKM cell culture (Fig. 5). Conversely, the Kenya05/Tk-1 strain, which was described as a moderate virulent strain^22^, showed large plaque phenotype while the Armenia07 strain, the most virulent among these three strains, exhibited intermediate plaque phenotype (Fig. 5). Considering that the Kenya05/Tk-1 and the Armenia07 strains showed similar replication profiles in the IPKM cell cultures (Fig. 4), replication rate did not directly link to neither plaque phenotype in vitro nor virulence of the virus in vivo. Transmission efficiency and capacity of host immune evasion may be related with the plaque phenotype by affecting the spread of the virus existing in infected cells^21^. Currently, viral factor(s) that governs the plaque phenotype remains unknown. As we described in this report, ASFV adopted to the IPKM cell culture retained only minor genetic mutations and was likely to maintain primary characteristics of ASFV isolates during at least 15 rounds of passage (Table1). Thus, the IPKM cell culture system would provide a useful platform to investigate the relation between viral characteristics and fitness.

Sánchez et al*.* reported that the immortalized porcine cell lines, a porcine alveolar macrophage-derived cell line (IPAM) and a wild boar lung-derived cell line (WSL), were susceptible to the naturally attenuated strain, NHV/P68 (genotype I), but not to virulent ones, i.e., Armenia07 and E70 strains (genotype I)^23^. These cell lines have also been reported to be sensitive to only limited strains of ASFV^24–26^. In this report, we demonstrated that the IPKM cell line is susceptible to virulent strains of different ASFV genotypes, Armenia07, Kenya05/Tk-1 and Espana75, as well as an artificially attenuated strain, Lisbon60V (Fig. 4). These findings suggested that, unlike IPAM and WSL, IPKM cells might be susceptible to various ASFV isolates, regardless of the virulence, genotype, or adaptation to other non-host cell cultures. Recently, a porcine macrophage cell line, Zuckerman macrophage-4 (ZMAC-4), which was susceptible to infection of eight different ASFV field isolates and supported their growth with similar kinetics which were observed in primary porcine macrophage cultures, was reported^27^. Although the virus developed HAD in the ZAMC-4 cell culture, however, it did not develop CPEs after infection. In contrast, IPKM cells grow in a single layer at the bottom of a cell culture plate and show marked CPEs. Therefore, the IPKM cell culture system has advantage over the ZAMC-4 system in isolating ASFV by plaque forming assays. The plaques can also be visualized by biological staining with neutral red, allowing to harvest viruses intact. By using this technique, we successfully isolated similar but significantly different clones from a pork meat product that contained a mixture of ASFVs^28^. This finding indicates that IPKM cell-based isolation may be valuable for purifying virus clones with high resolution.

The replication profiles of ASFV isolates observed in IPKM cultures were comparable to those in PAM cultures, although the growth of the viruses seemed faster in IPKM cultures than in PAM cultures, especially at the early to middle stages of infection (Fig. 4). These results are crucial for the quick preparation of ASFV quality stocks for genetic, biological and pathological studies of the virus. Furthermore, these features of IPKM cells are appropriate for applied research, such as the large-scale production of diagnostic reagents, anti-viral drug screening or the development of effective vaccines in the future. However, it remains unclear that the replication of ASFVs in the IPKM cell culture is faster than that in the PAM cell cultures. The IPKM cells and PAM cells were originated from different tissues, hence, both macrophage cells may differ in virus uptake, antiviral responses against ASFV, etc., resulting in the variability of viral replication efficiency. Alternatively, the numbers of ASFV particles released from the PAM cells may be lower than that from the IPKM cells in the early stages of infection, even though both cells support viral replication with the same efficiency. Comparing the viral titers in total of culture supernatants and cell lysates after ASFV infection over time may answer the question.

To our best knowledge, the IPKM cells supported the viral replication of virulent field isolates more efficiently than other reported cell lines. It was previously suggested that the expression level of CD163, a surface marker for mature macrophages, may be associated with the susceptibility of cells to ASFV in vitro^13^. In a previous report, however, we demonstrated that the IPKM cells expressed macrophage-specific surface marker proteins, Iba1, KT022, and Cd172a at high levels, but not CD163^14,29^. Our findings are consistent with the results of recent studies, which indicated that CD163 was not essential for ASFV infection^30,31^. In addition, WSL and IPAM, different macrophage cell lines showed the susceptibility to ASFVs, although it expressed a minimal level of CD163^23^. The key factor(s) required for virulent virus propagation remains for further investigation.

It is also important to note the frequency of genetic alterations in the viral genome, which may occur during repeated passages in IPKM cell cultures. Previous reports have demonstrated that some field ASFV isolates could be adapted to in vitro cell culture systems of non-host cells, for example, Vero and MS (monkey stable cell line) cells, which are African green monkey kidney-derived cell lines^24,25,32^. However, the viruses that were adapted to these cells frequently have notable deletions in their genomes, especially in the variable regions at both ends^16–19^. In contrast to these previous findings, we demonstrated that IPKM cells could support efficient and consistent propagation of ASFV without showing a significant increase in the rate of spontaneous mutation (Table 1). This result strongly suggests that the repetitive passage of ASFV isolates in IPKM cell cultures may have only a limited effect on their virulence as that in primary cell cultures. Analysis of genetic alterations accumulating in the ASFV genome in correlation with the increase of the number of passages in the IPKM cell culture has currently been underway.

In conclusion, we demonstrated that IPKM, an immortalized porcine macrophage cell line, is highly susceptible to ASFV infection and supports the propagation of both virulent and cell-adapted isolates of ASFV, including the Armenia07 strain, which is currently circulating in Europe and Asia–Pacific regions. This cell line can easily be maintained at a laboratory and provides many valuable features appropriate for the isolation, replication, and manipulation of ASFV. Hence, IPKM cells will be a powerful tool to further our knowledge of ASFV and promote future advances in the development of novel technologies to combat ASF, such as live vaccines.

## Methods

### Ethics statement

Animal experiment procedures were carried out in compliance with the regulations outlined in *Guide for the Care and Use of Laboratory Animals* of the National Institute of Animal Health (NIAH), National Agriculture and Food Research Organization (NARO), *Guidelines for Proper Conduct of Animal Experiments* of the Science Council of Japan^33^ and the ARRIVE guidelines^34^. The animal study was reviewed and approved by the Institutional Animal Care and Use Committee at the NIAH, NARO (approval number 20-046).

### Cells

The IPKM cell line was established as described previously^12^. Briefly, recombinant lentivirus vectors, pLVSIN-EF1α neo (Takara Bio Inc., Japan) encoding SV40LT and pTERT were inoculated into primary cultures of kidney macrophages collected from a 6-day old pig in the presence of 8 μg/mL polybrene (Sigma, USA). When proliferating cells appeared, 800 μg/mL G418 (Thermo Fisher Scientific, USA) was added for the antibiotic selection and further cultured for several weeks. The established cell line (IPKM) was routinely maintained in Dulbecco’s modified Eagle’s medium (DMEM, Nakarai Tesque, Japan) supplemented with 10% fetal bovine serum (FBS), 10 μg/mL bovine insulin (Sigma), 25 μM monothioglycerol (Wako, Japan), and antibiotics in cell culture plates and flasks for suspension culture (Sumitomo Bakelite Co., Japan).

PAM cells were prepared from 8-week old Landrace-Large White-Duroc pigs, as described previously^24^. The cells were cultured in RPMI1640 (Nacalai Tesque) containing 10% FBS and antibiotics at 37 °C in a 5% CO_2_ (95% air) incubator.

### Viruses

The virulent ASFV field isolates, Armenia07 (epidemic strain, genotype II), Espana75 (genotype I), and the moderately virulent ASFV field isolate Kenya05/Tk-1 (isolated from soft tick, genotype X) were courteously provided by Dr. Sanchez-Vizcaino (Universidad Complutense de Madrid, Madrid, Spain). These isolates had no history of passage in or adaptation to established cell lines and were routinely maintained in PAM cell cultures and stored in aliquots at − 80 °C until use. The Lisbon60 (genotype I) isolate was kindly provided by Dr. Genovesi (Plum Island Animal Disease Center, USA). To establish the Vero cell-adapted viruses, the parental isolate, which was propagated in PAM cell cultures, was serially passaged in Vero cell cultures. In the present study, we used the Vero cell-adapted virus, Lisbon60V, at a passage level of 33. All the experiments with ASFV were performed in the biosafety level 3 facility of the NIAH approval of the national authority of Japan.

### Virus titration

Viral titers of supernatants from cell culture inoculated with ASFV isolates were determined for PAM and IPKM cell cultures. PAM cells (1 × 10^5^ cells/mL) and IPKM cells (1 × 10^4^ cells/well) were seeded in each well of a 96-well cell culture plate, 2–3 days before the assay. One hundred microliters of tenfold serially diluted samples were inoculated into the wells in quadruplicate and incubated for 7 days at 37 °C in a 5% CO_2_ (95% air) incubator. The presence of CPEs was examined by microscopy, and the 50% tissue culture infectious dose per mL of each sample (TCID_50_/mL) was calculated using the Reed and Muench’s method^35^.

Additionally, we performed HAD assays using porcine red blood cells to detect the presence of the virus or to determine the viral titers of the tested samples. Twenty microliters of 0.75% (v/v) suspension of fresh procine red blood cells were added to the ASFV-infected host cell cultures in each well of a 96-well cell culture plate on the day of inoculation. The cultures were incubated for 7 days at 37 °C in a 5% CO_2_ (95% air) incubator and examined for the presence of rosette formation by microscopy. Viral titers were calculated as HAD units yielding 50% of cumulative infection per mL (HAD_50_/mL). The titers of the inoculates used in the immunofluorescence assays (Fig. 1) and the virus propagation assays (Fig. 4) were determined using this method in PAM cells.

### Immunofluorescence assay

The IPKM cells (2 × 10^5^ cells/well) were seeded in each well of a 4-well Lab-Tek II chambered cover glass (Thermo Fisher Scientific). The cells were inoculated with ASFV isolates (Armenia07, Kenya05/Tk-1 and Espana75) at an MOI of 0.1, followed by incubation for 16 h at 37 °C in a 5% CO_2_ (95% air) incubator. After washing once with phosphate-buffered saline (PBS), the cells were fixed with 80% acetone for 10 min on ice. The fixed cells were then treated with fluorescein isothiocyanate (FITC)-conjugated anti-ASFV antibodies^36^, which is recognized the structure protein of ASFV, for 1 h at ambient temperature. Fluorescence signals were observed under a fluorescence microscope (LSM 700, Carl Zeiss, Switzerland).

### Plaque forming assay

The IPKM cells (2 × 10^5^ cells/well) were cultured to confluence in 24-well cell culture plates. The cells were inoculated with 150 μL of culture supernatant inoculated with ASFV isolates (Armenia07, Kenya05/Tk-1 and Espana75) prepared by tenfold serial dilution with culture medium, and incubated for 1 h at 37 °C with tilting at 15 min intervals. After the inoculum was removed, 1 mL of the IPKM growth medium containing 1% SeaPlaque agarose (Lonza, Switzerland) was added into each well and left for 20 min at ambient temperature for solidification. One milliliter of the medium was then overlaid on each well, and incubated for 7 days at 37 °C in a 5% CO_2_ (95% air) incubator. Following incubation, the cells inoculated with ASFV isolates (Armenia07, Kenya05/Tk-1 and Espana75) were stained with 0.1% crystal violet in 5% formaldehyde to visualize plaques.

### Virus propagation

PAM cells (7.5 × 10^5^ cells/well) and IPKM cells (3 × 10^5^ cells/well) were dispersed in 24-well cell culture plates and incubated for 2 days. The cells were then inoculated with ASFV isolates (Armenia07, Kenya05/Tk-1, Espana75 and Lisbon60V) at a MOI of 0.001. After incubation for 1 h at 37 °C with tilting at 15 min intervals, the inoculum was removed, and the cells were washed three times with PBS. Culture medium (500 μL) was then added to each well, and the cultures were incubated at 37 °C in a 5% CO_2_ (95% air) incubator. The culture supernatants were collected at 0, 24, 48, 72 h, and 96 hpi, and stored at − 80 °C until use. Viral titers were examined in the IPKM cell cultures and calculated as TCID_50_/mL, as described above.

### Statistical analysis

The Student’s *t*-tests were applied on paired data to determine statistical significance. Differences with *P* < 0.05 were considered significant. Statistical analysis was performed using KaleidaGraph software (Synergy Software, Reading, PA, USA).

### Next-generation sequencing of ASFV genomes

The ASFV Armenia07 isolate was repetitively propagated in IPKM cell cultures, as described above. To prepare nucleic acid samples for next-generation sequencing, culture supernatants containing ASFV were centrifuged at 174,699 × *g* at 4 °C for 3 h. The pellets were then resuspended in 100 µL of PBS and treated with 250 U of benzonase nuclease (Sigma, USA) at 37 °C for 1 h. The viral DNA was then extracted using a High Pure Viral Nucleic Acid Kit (Roche Diagnostics, Japan) and next generation sequencing was performed using Ion PGM™ (Thermo Fisher Scientific) according to the manufacturer’s protocols. The viral reads were trimmed and mapped to the ASFV Georgia 2007/1 isolate (GenBank accession no. FR682468), which were performed using the Galaxy web platform^37^.

## Data Availability

The data supporting the findings of this study are available within the paper. Source files for the microscopy images or the next-generation sequencing are available upon request.
